# Evidence that implementation intentions reduce self‐harm in the community

**DOI:** 10.1111/bjhp.12682

**Published:** 2023-08-07

**Authors:** Abigail Paterson, Mark A. Elliott, Louise A. Brown Nicholls, Susan Rasmussen

**Affiliations:** ^1^ School of Psychological Sciences and Health University of Strathclyde Glasgow UK

**Keywords:** goal intention, implementation intention intervention, mental imagery, self‐harm, volitional help sheet

## Abstract

**Objectives:**

Implementation intentions are ‘IF‐THEN’ plans that encourage goal‐intended behaviour. This study was designed to test whether an intervention encouraging the formation of implementation intentions can reduce self‐harm in the community.

**Design:**

A randomized controlled design was used.

**Methods:**

At pre‐intervention, outcome variables (self‐harm in both specified and unspecified critical situations and suicidality) and potential moderators of implementation intentions (goal intention, mental imagery, and exposure to self‐harm) were measured using self‐report questionnaires. The participants (*N* = 469, aged 18–66 years, 86.4% female, 6.8% male and 6.7% other) were then randomized to either an experimental (implementation intention) or control task. At three‐months post‐intervention, self‐report questionnaires were used again to measure the outcome variables.

**Results:**

There were no overall differences between the conditions at post‐intervention. However, goal intention and mental imagery, but not exposure to self‐harm, moderated the effects of condition on self‐harm in specified critical situations. At high (mean + 1*SD*) levels of both goal intention and mental imagery, the experimental condition reported self‐harming less frequently in the situations specified in their implementation intentions.

**Conclusions:**

Implementation intentions therefore represent a useful intervention for reducing self‐harm in specified critical situations for people in the community who wish to avoid self‐harm and those who frequently experience self‐harm and suicide related mental imagery.

**Statement of contribution**

***What is already known on this subject?***

Implementation intentions are an effective behaviour change strategy for a range of health and social behaviours (Gollwitzer & Sheeran, 2006). This intervention has shown promise for reducing self‐harm related outcomes in patients admitted to hospital for self‐harm (Armitage et al., 2016; O'Connor et al., 2017). However, self‐harm is a largely hidden behaviour meaning that many people who engage in self‐harm do not present to hospital. It is important, therefore, to test interventions in the wider community, in addition to hospital patients.

***What does this study add?***

- Tests the effectiveness of implementation intentions at reducing self‐harm in a community sample.
- Tests whether goal intention, mental imagery or exposure to self‐harm moderate the intervention.

## INTRODUCTION

In the UK, self‐harm has been defined as the “intentional self‐poisoning or injury irrespective of the apparent purpose of the act” (NICE, 2022, p. 46). It is one of the strongest predictors of future suicide (Geulayov et al., 2019), it puts large demands on hospital services (Hawton et al., 2007), and it has economic consequences relating to health and social care (Tsiachristas et al., 2020). Psychological interventions to treat self‐harm typically focus on addressing people's reasons for this behaviour (Gibson et al., 2014). These interventions are often tested using hospital patients (Witt et al., 2021) rather than people in the community (i.e., people who self‐harm but do not necessarily present to hospital). However, the reasons for self‐harm tend to differ between hospital and community populations. Self‐harm in hospital patients tends to be primarily underpinned by ‘a wish to die’ (de Beurs et al., 2018) whereas self‐harm in the community tends to be underpinned by a broader range of reasons including emotional distress, wanting to punish oneself and interpersonal problems (Hambleton et al., 2020; Rasmussen et al., 2016). In addition, self‐harm in the community is approximately 10 times more prevalent than in people who present to hospital (Geulayov et al., 2018). People who present to hospital also tend to have a history of self‐harm prior to their first hospital admission (Mars et al., 2019). It is important, therefore, to test interventions in the wider community, in addition to hospital patients, to ensure they are effective at managing the wider range of reasons for self‐harm, and to reduce the number of future hospital presentations (i.e., to aid early intervention).

Psychological interventions for self‐harm (e.g., talking therapies) require face‐to‐face contact with a health professional. Many people avoid these interventions due to feelings of shame and stigma associated with self‐harm (Long, 2018). Brief interventions, which involve minimal contact with a health professional (Werch et al., 2006), are therefore likely to constitute a desirable method for reducing self‐harm. In addition, they are cost‐effective and capable of reaching large sections of the population (i.e., Duhem et al., 2018). However, they are typically not based on theory and there is limited evidence that they reduce self‐harm (Milner et al., 2015). In this study, we therefore tested a brief, theory‐based intervention to reduce self‐harm in the community.

The intervention tested in this study was based on Gollwitzer's (1993) concept of implementation intentions. Implementation intentions are IF‐THEN plans that promote goal‐intended behaviour. They require people to specify a critical situation (e.g., “If I am tempted to self‐harm when I feel hopeless…”) and link it with a goal‐directed response (e.g., “…then I will contact a helpline or a self‐harm support group”). This process generates a mental representation of the critical situation and its link to the goal‐directed response. When the critical situation is subsequently encountered, the mental representation is “activated”. This makes the encountered situation salient, increasing the likelihood that an opportunity to carry out the goal‐intended behaviour is not missed. It also promotes a suitable strategy that helps ensure the goal‐intended behaviour is performed (Webb & Sheeran, 2007).

There are two key reasons for expecting implementation intentions to be effective at reducing self‐harm. First, sample means on measures of goal intentions to self‐harm tend to be between the bottom and middle of the measurement scales (Armitage et al., 2016; O'Connor & Armitage, 2003), indicating that the avoidance of self‐harm is a goal intended behaviour for many people. Second, self‐harm is potentially influenced, or ‘triggered’, by many factors (Townsend et al., 2016). These triggers (e.g., feelings of hopelessness or entrapment) represent critical situations that increase the likelihood of self‐harm and implementation intentions provide strategies (goal‐directed responses) for coping with critical situations.

Implementation intentions have been found to change many behaviours (Gollwitzer & Sheeran, 2006). In the present context, they have also been found to be effective at improving outcomes for people admitted to hospital following self‐harm. Armitage et al. (2016), found that they reduced measures of suicidality (i.e., measures based on suicidal intent) at 3‐ and 6‐month post‐intervention. O'Connor et al. (2017) found that they reduced hospital re‐presentations at 6‐month post‐intervention for patients with at least one hospitalization for self‐harm in the last 10 years. Despite these promising results, no studies have tested whether implementation intentions can reduce self‐harm in the wider community. The primary aim of this study, therefore, was to address this gap.

This study also differed from the above cited research in two key ways. First, the primary outcome measure was the frequency of self‐harm behaviour in the critical situations specified in the IF components of participants' implementation intentions. Measures of suicidality (Armitage et al., 2016) and hospital re‐admissions (O'Connor et al., 2017) are likely to constitute appropriate outcome measures for research with hospital patients because a key motive for self‐harm within this group is the wish to die (de Beurs et al., 2018). As a result, a preponderance of hospital patients present with suicidal intent (Cleare et al., 2021; de Beurs et al., 2018). It is reasonable, therefore, to expect reductions in suicidality and readmission to hospital to be observed following a self‐harm intervention. However, the proportion of people in the wider community classified as being suicidal tends to be low (Fitzpatrick et al., 2020; Osman et al., 2001; Robinson et al., 2021) and a direct measure of self‐harm frequency ensures that the wider range of motives for self‐harm that are typically found in the community (McManus et al., 2019; Rasmussen et al., 2016) are captured, thus reducing the risk of unduly underestimating intervention effects. Furthermore, focusing on the frequency of self‐harm in the critical situations that are specified in the IF components of participants' implementation intentions ensured that the primary outcome measure aligned with the theoretical proposition that implementation intentions generate behaviour change when people encounter the salient features of the specified critical situations (Bieleke et al., 2021). Following recent research on other health‐related behaviours supporting this theoretical proposition (Brewster et al., 2016; Elliott et al., 2021), the primary outcome measure in this research was the frequency of self‐harm in the critical situations that the participants specified in the IF component of their implementation intentions.

Second, this study differed from previous research with hospital patients because we tested key moderators of implementation intentions: goal intentions, mental imagery and exposure to self‐harm. As already mentioned, implementation intentions generate changes in goal‐intended behaviour, meaning that an individual must already possess the required goal intention (i.e., to avoid self‐harm) before an implementation intention intervention will evoke behaviour‐change (Gollwitzer, 1993). Accordingly, goal intentions have been found to moderate the effects of implementation intentions in several studies of other behaviours, outside the context of self‐harm, with behaviour‐change occurring more readily at high (mean + 1*SD*), versus low (mean − 1*SD*), pre‐intervention levels of goal intentions (Elliott & Armitage, 2006; Sheeran et al., 2005 [study 1]).

Mental imagery and exposure are important determinants of self‐harm and suicide in the Integrated Motivational‐Volitional Model of Suicidal Behaviour (IMV; O'Connor & Kirtley, 2018). Mental imagery refers to the frequency with which people experience suicide‐related images, including images of self‐harm. Exposure is typically operationalized as the extent to which people have been exposed to significant others (e.g., family or friends) who engage in this behaviour. Research has shown that mental imagery and exposure both increase the likelihood of self‐harm (Branley‐Bell et al., 2019). Individuals who experience high levels of these constructs may therefore have difficulties regulating their self‐harm behaviour and therefore benefit from an implementation intention intervention, as it provides self‐regulatory strategies (i.e., goal‐directed responses for managing the critical situations that trigger self‐harm). On the other hand, individuals with low levels of mental imagery or exposure are likely to find it easier to regulate their behaviour and therefore benefit less from an implementation intention intervention.

### Aims and hypotheses

This study was designed to test the effects of an implementation intention intervention on self‐harm behaviour in a community sample. We hypothesized that self‐harm in specified critical situations would be reported less frequently by the participants randomly assigned to an experimental (implementation intention) condition than those randomly assigned to a control condition (hypothesis 1). We also hypothesized that this main effect would be moderated by goal intention (hypothesis 2), mental imagery (hypothesis 3), and exposure to self‐harm (hypothesis 4), with implementation intentions reducing self‐harm at high (mean + 1*SD*), but not low (mean − 1*SD*) levels of these moderators. We also tested these main and moderator effects using measures of self‐harm in unspecified critical situations, to gauge whether there may be any spill‐over effects (i.e., from specified to unspecified critical situations). Following Armitage et al. (2016), the main and moderator effects were also tested using measures of suicidality to determine whether any reductions in self‐harm translated into reductions in suicidal thoughts and behaviour, consistent with research with hospital patients.

## METHOD

### Participants

The sample comprised 469 participants. All participants had reported self‐harming in the three months prior to the study. The socio‐demographic profile is shown in Table 1. These data are broadly consistent with previous research on self‐harm (Branley‐Bell et al., 2019). Power analysis indicated that a final sample of *n* = 259 was required to detect *d* = .35, at alpha = .05 and power = .80 (Cohen, 1992). Given the achieved sample exceeded the target sample, it was deemed that the analyses were appropriately powered.

**TABLE 1:** Socio‐demographic characteristics of the sample.

Demographic characteristic	*n*	%
Gender		
Female	405	86.4
Male	32	6.8
Transgender (male to female)	3	.6
Transgender (female to male)	11	2.3
Transgender (do not identify as male or female)	9	1.9
Not sure	7	1.5
Declined to state	2	.4
Marital status		
Married	27	5.8
In a relationship, but not married	181	38.6
Single	250	53.3
Separated	3	.6
Divorced	5	1.1
Widowed	3	.6
Employment status		
Full‐time paid work	101	21.5
Part‐time paid work	56	11.9
Full‐time voluntary (unpaid) work	2	.4
Part‐time voluntary (unpaid) work	7	1.5
Full‐time education	219	46.7
Part‐time education	17	3.6
Homemaker	11	2.3
Unemployed	56	11.9
UK resident		
Yes	324	72.6
No	122	27.4

*Note*: *N* = 469. Participants were on average 23.09 years old (*SD* = 6.76).

### Design and procedure

A randomized‐controlled design was used. Advertisements were placed on social media (e.g., Facebook, Twitter, Reddit), online recruitment platforms and forums (e.g., Call for Participants, National Self‐Harm Network, Postgraduate JiscMail) and a participation pool at the authors' institution. The advertisement stated that the study was being conducted to understand self‐harm in the general population. It asked for volunteers who were aged 18+ years old and had self‐harmed in the three months prior to the study. Potential participants were asked to click on a link within the advertisement, which directed them to an online pre‐intervention questionnaire.

The pre‐intervention questionnaire was developed and administered using Qualtrics (software for survey design and administration). It contained standard items to measure socio‐demographic variables (see Table 1), the outcome variables (frequency of self‐harm in both specified and unspecified critical situations and suicidality) and moderators (goal intention, mental imagery, and exposure to self‐harm). On completion of these items, Qualtrics automatically randomized the participants to an experimental or a control condition. The experimental condition was presented with an intervention that asked participants to link critical situations with goal‐directed responses to avoid self‐harm (i.e., form implementation intentions). The control condition was presented with an intervention that asked them to select critical situations and try to avoid self‐harm when they were encountered.

After three months, the participants were emailed a link to an online, post‐intervention questionnaire in Qualtrics. The post‐intervention questionnaire measured the outcome variables using the same items as the pre‐intervention questionnaire. The pre‐ and post‐intervention data were matched using unique codes that were generated from information provided in both questionnaires (the first initial of their first name, the last initial of their surname, the last two digits of their phone number and the last two letters of their postcode). Once the data were matched, the unique codes and any other identifying information (e.g., email addresses) were deleted to ensure anonymity. Ethical approval was granted by the University's Research Ethics Committee.^1^

### The implementation intention intervention

The implementation intention intervention was an online volitional help sheet (see Appendix S1). It provided participants with pre‐specified critical situations and goal‐directed responses and instructions on how to use these to form implementation intentions (Armitage, 2008). The participants were presented with a drop‐down list of 20 critical situations, formulated as IF statements (e.g., ‘IF I am tempted to self‐harm when I feel hopeless…’). The critical situations were triggers for self‐harm as indicated by people with lived experience as part of previous research. They were the same evidence‐based situations used by Armitage et al. (2016) and O'Connor et al.'s (2017) in their research on implementation intentions, but supplemented with additional situations from key, evidence‐based, theoretical frameworks that specify triggers for self‐harm and suicide (O'Connor & Kirtley, 2018). The participants were asked to choose the critical situation that they felt would tempt them the most to self‐harm over the next three months. Next, the participants were presented with a drop‐down list of 22 goal‐directed responses, formulated as THEN statements (e.g., ‘…THEN I will contact a helpline or a self‐harm support group’). These were also taken from the volitional help sheet developed by Armitage et al. (2016) and they were supplemented with strategies from self‐harm support resources which draw upon the lived experiences of people who self‐harm (Mind, 2020). Following previous studies (Armitage, 2008), the 22 goal‐directed responses mapped onto the 10 processes of behaviour‐change that are specified in the transtheoretical model (Prochaska et al., 1988). The participants were asked to select the goal‐directed response that would be most helpful for helping them avoid self‐harm should they find themselves in their chosen critical situation. Consistent with several studies (Elliott et al., 2021), this task was presented a further three times, thus encouraging the formation of four implementation intentions in total.

### The control intervention

An active control group was used to compensate for the possible demand placed on the experimental condition by the volitional help sheet (Rosenthal, 1966). The control intervention contained the same list of 20 critical situations that was used in the implementation intention intervention. However, the critical situations were presented as WHEN statements rather than IF statements (e.g., ‘I am most likely to self‐harm WHEN I feel hopeless’). Consistent with the approach used in several studies (Elliott et al., 2021) the control participants were asked to select the four critical situations that would tempt them the most to self‐harm. However, rather than link each critical situation with a goal‐directed response, and therefore form implementation intentions, they were asked to try to avoid self‐harming in their chosen critical situations over the next three months.

### The outcome variables

#### Self‐harm behaviour in specified and unspecified critical situations

The method used in previous studies for measuring behaviour in specified and unspecified critical situations was followed (Elliott et al., 2021). The participants were asked: ‘How many times, over the LAST 3 months, have you harmed yourself in the following situations?’ They were then presented with the 20 critical situations that were contained in the interventions. For each situation, the participants responded on a 9‐point scale (1 = *No times* to 9 = *Many times*). The mean of the items that corresponded to the four critical situations selected by the participants in the interventions was used as the final measure of self‐harm behaviour at both pre‐intervention (*α* = .90) and post‐intervention (*α* = .94). The final measures of self‐harm behaviour in unspecified critical situations were calculated by taking the means of the items that corresponded to the remaining, unchosen, situations (*α* = .91 at pre‐intervention and *α* = .94 at post‐intervention).

#### Suicidality

Following Armitage et al. (2016), the four items from the Suicide Behaviour Questionnaire‐Revised (SBQ‐R; Osman et al., 2001) were used as separate outcome measures (see Tables 2, 3, 4). These items have been combined into a single scale in several studies of self‐harm (Akram et al., 2020). However, this made no difference to the present findings.

**TABLE 2:** Analyses of variance (ANOVAs) testing pre‐intervention differences between dropouts versus study completers, and control versus experimental conditions.

Dependent variable	*F* (*df* = 1, 467)	*MSE*	*p*	*d*
Attrition (0 = Dropouts; 1 = Study Completers)				
Self‐harm in specified situations	.05	3.79	.823	−.02
Self‐harm in unspecified situations	5.85	3.00	.016	.22
SBQ‐R Suicidal ideation and behaviour	.61	2.03	.437	.07
SBQ‐R Frequency of suicidal thoughts	.07	2.03	.790	−.03
SBQ‐R Threat to die by suicide	.17	1.91	.682	.04
SBQ‐R Likelihood of a future suicide attempt	3.11	1.74	.079	.16
Goal intention	4.84	3.34	.028	−.20
Mental imagery	2.45	3.48	.119	.15
Exposure to friend self‐harm	10.88	5.64	.001	.31
Exposure to family self‐harm	.15	7.38	.702	.04
Condition (0 = Control; 1 = Experimental)				
Self‐harm in specified situations	.14	3.79	.707	−.03
Self‐harm in unspecified situations	1.23	3.03	.269	−.10
SBQ‐R Suicidal ideation and behaviour	.04	2.04	.833	−.02
SBQ‐R Frequency of suicidal thoughts	.08	2.03	.773	−.03
SBQ‐R Threat to die by suicide	.59	1.91	.443	−.07
SBQ‐R Likelihood of a future suicide attempt	.87	1.74	.352	.08
Goal intention	1.65	3.37	.199	−.12
Mental imagery	1.75	3.49	.186	.12
Exposure to friend self‐harm	.90	5.76	.343	.09
Exposure to family self‐harm	.92	7.37	.337	.09

Abbreviation: SBQ‐R, Items from the Suicide Behaviour Questionnaire‐Revised.

**TABLE 3:** Pre‐ and post‐intervention means and standard deviations, and one‐way Analyses of Covariance (ANCOVAs) to test the differences between the conditions.

	*M* (*SD*)				ANCOVA				
	Pre‐intervention		Post‐intervention		*F*	*df*	*MSE*	*p*	*d*
	Cont	Exp	Cont	Exp					
Self‐harm (specified situations)	7.27 (1.95)	7.34 (1.94)	6.71 (2.44)	6.53 (2.56)	2.47	1, 466	2.85	.116	.14
Self‐harm (unspecified situations)	4.57 (1.73)	4.75 (1.75)	4.28 (1.90)	4.37 (2.01)	.75	1, 466	1.06	.386	.08
SBQ‐R Suicidal ideation and behaviour	3.04 (1.40)	3.07 (1.45)	3.05 (1.49)	3.10 (1.47)	.09	1, 466	1.57	.766	−.02
SBQ‐R Frequency of suicidal thoughts	3.53 (1.41)	3.57 (1.44)	3.45 (1.44)	3.50 (1.44)	.05	1, 466	1.59	.830	−.02
SBQ‐R Threat to die by suicide	1.84 (1.35)	1.93 (1.42)	1.80 (1.32)	1.98 (1.40)	1.43	1, 466	1.31	.232	−.11
SBQ‐R Likelihood of a future suicide attempt	2.43 (1.35)	2.32 (1.29)	2.51 (1.45)	2.33 (1.34)	1.09	1, 466	1.27	.296	.10
Goal intention	5.76 (1.82)	5.98 (1.85)	n/a	n/a	n/a	n/a	n/a	n/a	n/a
Mental imagery	6.44 (1.84)	6.21 (1.89)	n/a	n/a	n/a	n/a	n/a	n/a	n/a
Exposure to friend self‐harm	3.18 (2.39)	2.97 (2.41)	n/a	n/a	n/a	n/a	n/a	n/a	n/a
Exposure to family self‐harm	2.61 (2.79)	2.38 (2.63)	n/a	n/a	n/a	n/a	n/a	n/a	n/a

Abbreviations: CONT, Control Condition; EXP, Experimental condition; SBQ‐R, Items from the Suicide Behaviour Questionnaire‐Revised.

**TABLE 4:** Multiple linear regressions testing moderation effects.

Variables	*R* ^2^	*F*	*β*	*d*
Regression 1: Predicting post‐intervention self‐harm in specified situations	.57	62.78		
Pre‐Intervention Self‐Harm in Specified Situations			.84***	1.28
Condition (0 = control; 1 = experimental)			−.17	−.10
Goal Intention			−.20***	−.32
Mental Imagery			.08	.16
Exposure (Friends)			.03	.08
Exposure (Family)			.03	.10
Condition × Goal Intention			−.22*	−.23
Condition × Mental Imagery			−.23**	−.24
Condition × Exposure (Friends)			.02	.02
Condition × Exposure (Family)			.05	.08
Regression 2: Predicting post‐intervention self‐harm in unspecified situations	.73	127.13		
Pre‐Intervention Self‐Harm in Unspecified Situations			.91***	1.54
Condition (0 = control; 1 = experimental)			−.05	−.05
Goal Intention			−.10***	−.32
Mental Imagery			.01	.02
Exposure (Friends)			.02	.07
Exposure (Family)			.01	.04
Condition × Goal Intention			−.13*	−.23
Condition × Mental Imagery			−.13*	−.23
Condition × Exposure (Friends)			.03	.06
Condition × Exposure (Family)			.02	.05
Regression 3: Predicting post‐intervention SBQ‐R suicidal ideation and behaviour	.27	18.70		
Pre‐Intervention SBQ‐R Suicidal Ideation and Behaviour			.55***	.93
Condition (0 = control; 1 = experimental)			.08	.03
Goal Intention			−.04	−.10
Mental Imagery			−.01	−.03
Exposure (Friends)			.00	.01
Exposure (Family)			−.01	−.04
Condition × Goal Intention			.04	.06
Condition × Mental Imagery			.06	.09
Condition × Exposure (Friends)			.01	.01
Condition × Exposure (Family)			−.02	−.03
Regression 4: Predicting post‐intervention SBQ‐R frequency of suicidal thoughts	.25	16.54		
Pre‐Intervention SBQ‐R Frequency of Suicidal Thoughts			.39***	.70
Condition (0 = control; 1 = experimental)			.06	.05
Goal Intention			−.09**	−.24
Mental Imagery			.08*	.21
Exposure (Friends)			−.02	−.06
Exposure (Family)			−.02	−.09
Condition × Goal Intention			.02	.02
Condition × Mental Imagery			.09	.13
Condition × Exposure (Friends)			−.01	−.03
Condition × Exposure (Family)			.01	.03
Regression 5: Predicting post‐intervention SBQ‐R threats to die by suicide	.29	19.75		
Pre‐Intervention *SBQ‐R T*hreats to Die by Suicide			.54***	1.06
Condition (0 = control; 1 = experimental)			.13	.11
Goal Intention			.00	.01
Mental Imagery			.00	.01
Exposure (Friends)			−.00	−.00
Exposure (Family)			−.01	−.03
Condition × Goal Intention			−.01	−.01
Condition × Mental Imagery			−.01	−.02
Condition × Exposure (Friends)			.01	.01
Condition × Exposure (Family)			.02	.05
Regression 6: Predicting post‐intervention SBQ‐R likelihood of future suicide attempt	.36	26.85		
Pre‐Intervention SBQ‐R Likelihood of Future Suicide Attempt			.61***	1.06
Condition (0 = control; 1 = experimental)			−.10	−.09
Goal Intention			−.07*	−.21
Mental Imagery			−.04	−.10
Exposure (Friends)			.01	.04
Exposure (Family)			.00	.02
Condition × Goal Intention			−.00	−.00
Condition × Mental Imagery			.02	.02
Condition × Exposure (Friends)			.08	.18
Condition × Exposure (Family)			.04	.09

*Note*: *p < .05. **p < .01. ***p < .001.

Abbreviation: SBQ‐R, Items from the Suicide Behaviour Questionnaire‐Revised.

### The moderator variables

#### Goal intention

Four standard items (Fishbein & Ajzen, 2010) were used to measure goal intentions (e.g., “To what extent do you intend to avoid harming yourself over the NEXT 3 months?”). These were measured on 9‐point scales (e.g., 1 = *No extent at all* to 9 = *A great extent*). The mean of the four items produced the final measure of goal intention to avoid self‐harm (*α* = .78).

#### Mental imagery

Six items from the modified version of the 21‐item Social Cognitions Interview (Holaday & Brausch, 2015) were used to measure mental imagery (e.g., “How often do you find yourself thinking about a time when you tried to hurt yourself in the past”). These were measured on 9‐point scales (1 = *Never* to 9 = *Often*). The final measure of mental imagery was calculated by taking the mean of the six items (*α* = .78).

#### Exposure to self‐harm

Two items identified from previous studies (O'Connor et al., 2012) were used to measure exposure to self‐harm in the last three months: “Has anyone among your close friends attempted suicide or harmed themselves?” and “Has anyone among your family attempted suicide or harmed themselves?” (both scored as 1 = *None of them* to 9 = *Many of them*). The two items were not correlated (*r* = −.02, *N* = 469, *p* = .610) so were treated as separate measures of exposure in the subsequent data analyses, consistent with previous research (O'Connor et al., 2012).

## RESULTS

### Response rates and tests of attrition

A total of 469 participants completed a pre‐intervention questionnaire (*n* = 230 in experimental condition and *n* = 239 in control condition) and 216 completed a post‐intervention questionnaire (*n* = 103 in the experimental condition and *n* = 113 in the control condition). This completion rate compares favourably with previous studies of implementation intentions (Armitage et al., 2016) and self‐harm more generally (Rasmussen et al., 2016).

Analyses of Variance (ANOVAs) were conducted to test for potential pre‐intervention differences between the study completers (*n* = 216) and dropouts (*n* = 253). The dependent variables were the outcome and moderator variables at pre‐intervention. The independent variable was attrition (0 = dropouts; 1 = study completers). As Table 2 shows, the only detectable differences between the study completers and dropouts were for the measures of self‐harm in unspecified critical situations, goal intention and exposure to self‐harm by friends. Compared with the dropouts, the study completers reported self‐harming less frequently in unspecified critical situations (*M* = 4.45, *SD* = 1.56 vs. *M* = 4.83, *SD* = 1.87), higher goal intention to avoid self‐harm (*M* = 6.07, *SD* = 1.66 vs. *M* = 5.70, *SD* = 1.96) and lower exposure to self‐harm by their friends (*M* = 2.69, *SD* = 2.14 vs. *M* = 3.42, *SD* = 2.56).

Given the detectable differences between the study completers and dropouts, the intention to treat procedure (Hollis & Campbell, 1999) was used in the subsequent data analyses. The participants who dropped out of the study at post‐intervention were treated as “no‐changers” by imputing their pre‐intervention scores into the post‐intervention measures. This is a standard technique that is commonly used in intervention research because it preserves the initial sample. This prevents the loss of power due to attrition and provides conservative estimates of intervention effects that are derived from the full sample, rather than a potentially biased sample of study completers only (Lachin, 2000; McCoy, 2017).

### Tests of randomization

ANOVAs were also conducted to test for potential differences at pre‐intervention between the experimental and control conditions. The dependent variables were the outcome and moderator variables at pre‐intervention. The independent variable was condition (0 = control; 1 = experimental). As Table 2 shows, there were no differences observed between the experimental and control groups therefore randomization to conditions was deemed to be successful.

### Descriptive statistics

Table 3 shows that the sample reported a moderate‐to‐high frequency of self‐harm in specified critical situations (i.e., the sample means on this measure were between the mid‐point [5] and top [9] of the scale). It also reported a moderate frequency of self‐harm in unspecified critical situations, moderate levels of suicidal ideation and behaviour and suicidal thoughts, infrequent threats to die by suicide, a low likelihood of a future suicide attempt, high levels of goal intention to avoid self‐harm, high levels of self‐harm and suicide‐related mental imagery, and low levels of exposure to self‐harm. It is noteworthy, that the mean for the pre‐intervention measure of self‐harm in specified situations was higher than was the mean for the pre‐intervention measure of self‐harm in unspecified situations. This difference was found to be statistically significant for the experimental condition, *t*(229) = 29.80, *p* < .001, *d* = 1.96, and control condition, *t*(238) = 29.73, *p* < .001, *d* = 1.92. This suggests that the participants in both conditions were selecting the critical situations that would most tempt them to engage in self‐harm, as instructed.

### The effect of implementation intentions on self‐harm in specified critical situations

An Analysis of Covariance (ANCOVA) was conducted to test whether the experimental condition reported self‐harming less frequently than the control condition at post‐intervention in specified critical situations (hypothesis 1). The dependent variable was self‐harm in specified critical situations at post‐intervention. The covariate was self‐harm in specified critical situations at pre‐intervention. The independent variable was condition. Contrary to hypothesis 1, there was no statistically significant difference between the conditions (Table 3).^2^

To test whether goal intention (hypothesis 2), mental imagery (hypothesis 3), and exposure to self‐harm (hypothesis 4), moderated the effect of implementation intentions on the frequency of self‐harm in specified critical situations, a moderated multiple regression was conducted with follow‐up simple slopes analyses of any significant interactions (Aiken & West, 1991). The dependent variable was self‐harm in specified critical situations at post‐intervention. The independent variables were self‐harm in specified situations at pre‐intervention, condition, the potential moderators and the two way‐interactions between condition and each potential moderator. Potential multicollinearity was reduced by centring the independent variables prior to the computation of the interactions (Aiken & West, 1991).

**FIGURE 1:**
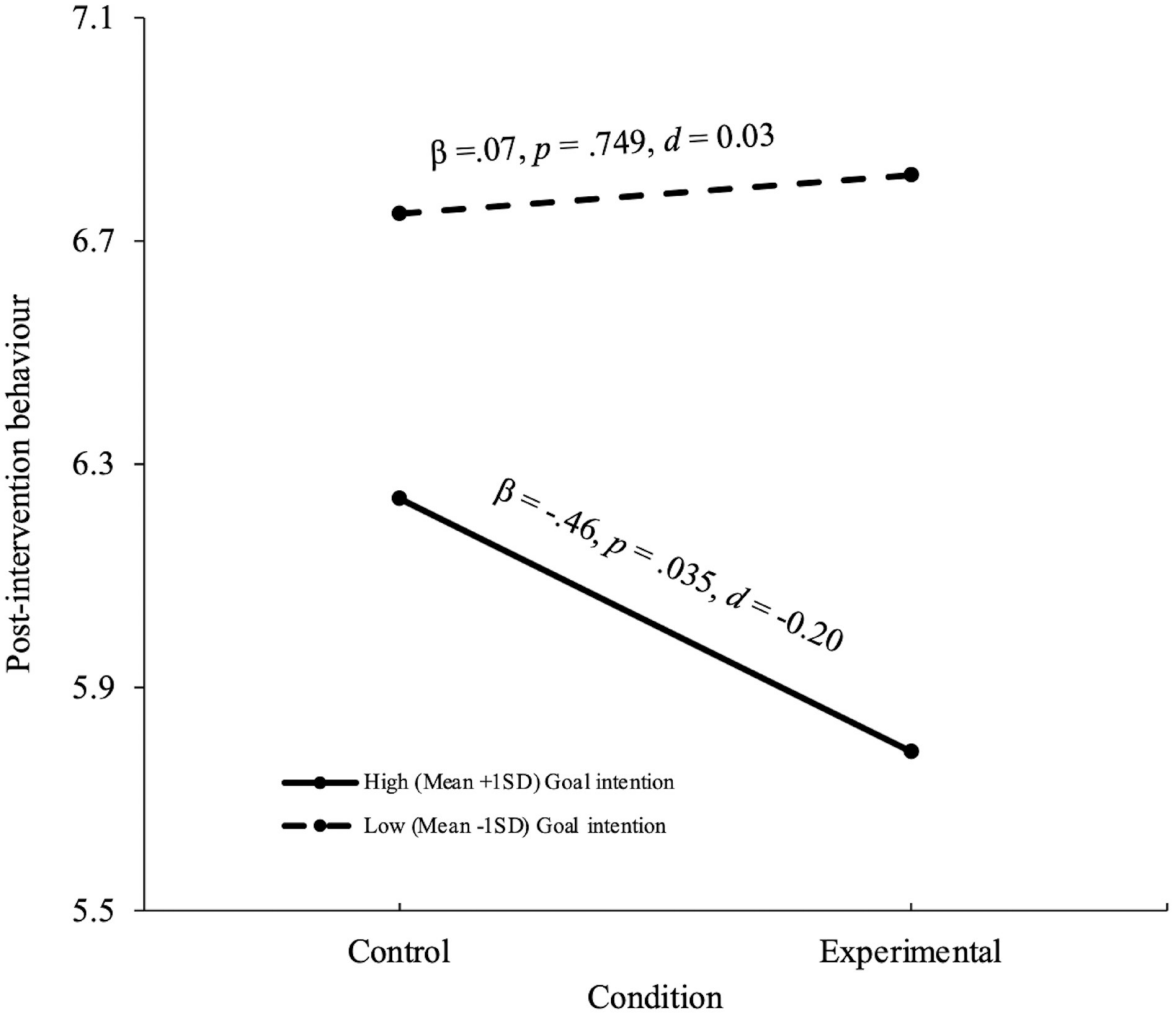
Simple slopes for condition at two levels of goal intention predicting post‐intervention self‐harm behaviour in specified critical situations.

**FIGURE 2:**
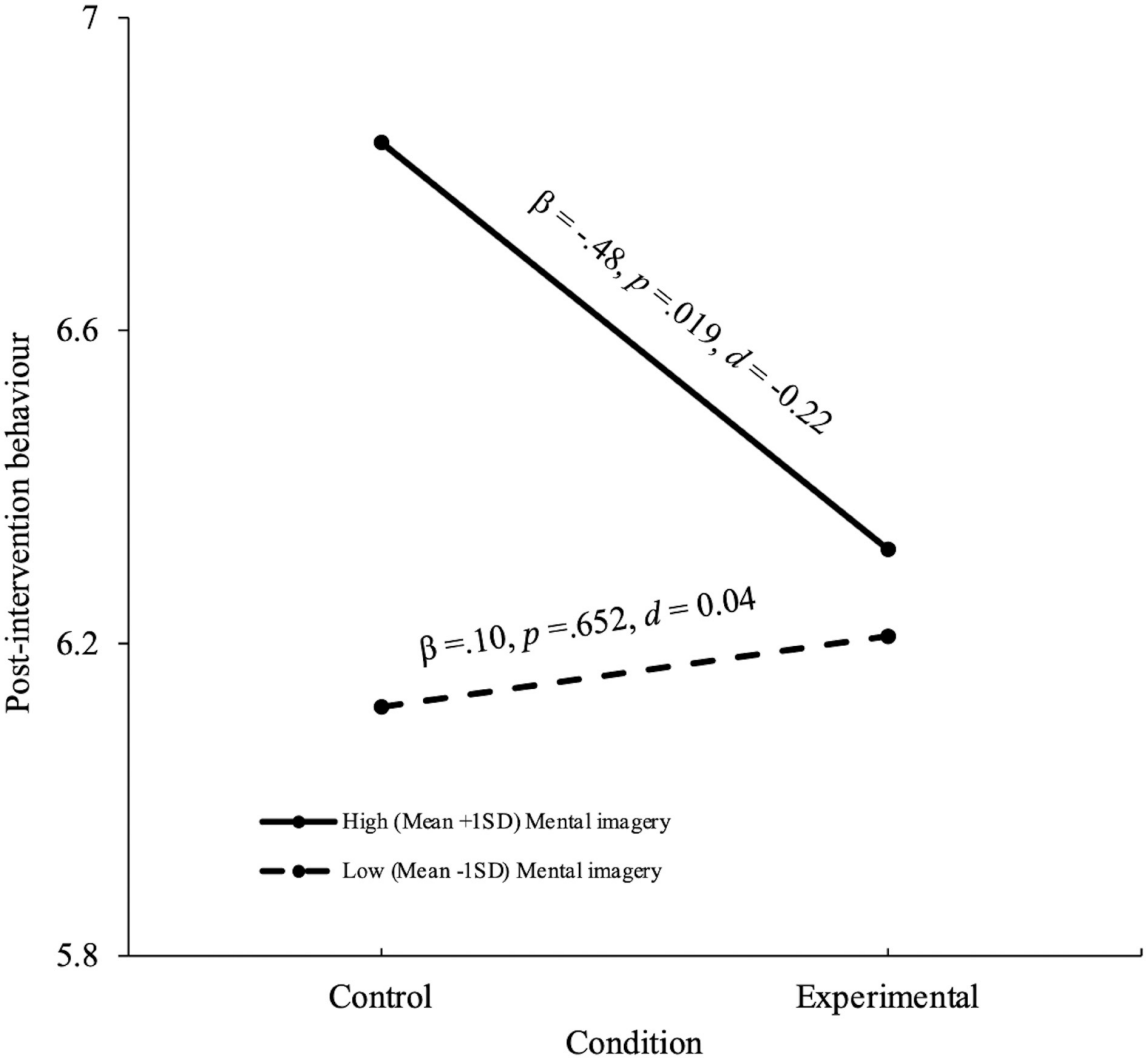
Simple slopes for condition at two levels of mental imagery predicting post‐intervention self‐harm behaviour in specified critical situations.

The regression accounted for 57% of the variance (Table 4). The condition × goal intention and condition × mental imagery interactions were independent predictors. Consistent with hypotheses 2 and 3, the follow‐up simple slopes analyses showed that the experimental condition reported lower levels of self‐harm in specified critical situations than did the control condition at high (mean + 1*SD*) but not at low (mean − 1*SD*) levels of goal intention (Figure 1) and mental imagery (Figure 2). Given that the two‐way interactions between condition and the two exposure to self‐harm measures were not statistically significant (Table 4), hypothesis 4 was not supported.

### The effect of implementation intentions on the other outcome variables

The same statistical procedures that were used to test the hypotheses were used to test the effects of implementation intentions on the other outcome variables. ANCOVAs showed that there were no differences between the conditions on the other outcome variables (Table 3). The regression analyses revealed that the only interaction effects were for condition × goal intention and condition × mental imagery in the prediction of self‐harm behaviour in unspecified critical situations (Table 4). However, simple slopes analyses showed that condition was not a statistically significant predictor at either high (mean + 1*SD*) or low (mean − 1*SD*) levels of goal intention (*β* = −.22, *p* = .103, *d* = −.15 and *β* = .11, *p* = .405, *d* = .08, respectively) or mental imagery (*β* = −.23, *p* = .070, *d* = −.17 and *β* = .10, *p* = .448, *d* = .07, respectively).

## DISCUSSION

This study was conducted to provide the first test of whether an implementation intention intervention can reduce self‐harm in the community. It was hypothesized that self‐harm in specified critical situations would be reported less frequently by the experimental (implementation intention) condition than the control condition (hypothesis 1). It was also hypothesized that a lower frequency of post‐intervention self‐harm in specified critical situations would be observed in the experimental (vs. control) condition at high, but not low, levels of goal intention (hypothesis 2), self‐harm and suicide‐related mental imagery (hypothesis 3), and exposure to self‐harm (hypothesis 4). The main and moderator effects were also tested using measures of self‐harm in unspecified critical situations and suicidality to explore potential spill‐over effects.

### The effect of implementation intentions on self‐harm and the other outcomes

Contrary to hypothesis 1, no overall difference was found between the experimental and control conditions in the frequency of self‐harm behaviour in specified critical situations at 3‐month post‐intervention. However, as expected, there was evidence that goal intentions and mental imagery moderated the implementation intention intervention. In support of hypothesis 2, the experimental condition reported self‐harming less often in specified critical situations than did the control condition at high (mean + 1*SD*) but not low (mean − 1*SD*) levels of goal intention to avoid self‐harm. The effect size was *d* = −.20. While this is recognized as a small effect size (Cohen, 1992), it is within the range of effect sizes that have been found in previous studies of implementation intentions for other social behaviours (Gollwitzer & Sheeran, 2006) and self‐harm in people who present to hospital (Armitage et al., 2016). This finding is also in line with Gollwitzer's (1993) theoretical proposition that implementation intentions promote goal‐intended behaviour because they generated behaviour‐change for people with the relevant goal intention (also see Elliott & Armitage, 2006; Sheeran et al., 2005 [study 1]). In the present context, this finding demonstrates, for the first time, that implementation intentions can reduce self‐harm in the community generally for those people who are theoretically appropriate to target with this type of intervention (i.e., those with the necessary goal intention to avoid self‐harm).

In support of hypothesis 3, the experimental condition also reported self‐harming less frequently than did the control condition at high, but not low levels of mental imagery (*d* = −.22). Although exposure did not moderate the effects of implementation intentions, in contrast to hypothesis 4, the findings provide some evidence that implementation intention interventions can change the behaviour of those individuals who are at greatest risk of self‐harm and who are likely to have the most difficulty regulating their behaviour. The present findings therefore attest to the efficacy of implementation intentions (Gollwitzer & Sheeran, 2006).

For the other outcome measures, there were no effects of implementation intentions, overall or at any level of the moderators. These results imply that the observed reductions in self‐harm behaviour in specified critical situations (at high levels of goal intention and mental imagery) do not generalize to reductions in self‐harm in unspecified situations. While this finding is consistent with some studies (Elliott et al., 2021; Webb & Sheeran, 2007), other studies have shown that implementation intentions generate behaviour‐change in unspecified critical situations to the extent that those unspecified critical situations share enough salient features with the situations that are specified (Brewster et al., 2016). Future research could test the extent to which implementation intention interventions can produce reductions in self‐harm in contextually similar critical situations to those specified by the participants.

The null results on the additional outcome measures also imply that implementation intentions are unable to reduce suicidality outcomes in a general community sample. This is not consistent with Armitage et al. (2016) who administered a similar implementation intention intervention, but with patients admitted to hospital for self‐harm. It is possible that implementation intentions to reduce self‐harm are better equipped to reduce measures of suicidality in hospital patients compared with the community because suicidal intent is more common for people who are admitted to hospital after self‐harm (Hawton et al., 2009). Indeed, mean scores on the suicidality outcomes are higher in suicidal sub‐groups (Osman et al., 2001). In accordance with this reasoning, the means on the suicidality outcomes in this study tended to be low, suggesting little scope for reductions.

### Practical implications

The findings imply that implementation intention interventions are likely to be useful for reducing self‐harm in the community for those who possess the required goal intention (i.e., to avoid harming themselves). Such individuals are likely to be prevalent in the population. As mentioned in the introduction, research suggests that the avoidance of self‐harm is goal‐intended for many individuals (Armitage et al., 2016; O'Connor & Armitage, 2003), which is consistent with the findings of this study as the sample means indicated an overall orientation towards the avoidance of self‐harm. Also, in addition to the findings reported in the results section, 15% of the sample in this study scored above the mean + 1*SD* on the measure of goal intention (i.e., the level at which the experimental condition was found to report a significantly lower frequency of post‐intervention self‐harm than the control condition). Implementation intentions are therefore likely to benefit hundreds of thousands of people in the UK alone, given that an estimated 380,000 adults in Scotland (Scottish Government, 2020) and 3.92 million adults in England (McManus et al., 2016) have ever reported harming themselves. In addition, reducing self‐harm behaviour for these individuals is likely to reduce future cases of hospitalizations and suicide (Geulayov et al., 2018) and other social and economic consequences of self‐harm (Tsiachristas et al., 2020).

The main advantage of brief interventions, such as the one tested in this study, is that they can be self‐completed, making them cost‐effective and easy to administer on a large scale. The present intervention could therefore be incorporated into existing efforts to tackle self‐harm in the community. It could be administered in self‐harm support groups (Boyce et al., 2018) or web‐based applications for self‐harm (Cliffe et al., 2021). This would be beneficial for reaching individuals in the community given that many do not seek medical or psychological treatment for their self‐harm and therefore do not see a health practitioner (McManus et al., 2019).

An additional advantage of an implementation intention intervention (e.g., the volitional help sheet used in this study) is that it emphasizes the importance of linking a critical situation with a goal‐directed response. This practice could be incorporated into safety planning interventions that are administered to people who self‐harm (Stanley & Brown, 2012), including the Attempted Suicide Short Intervention Programme (Gysin‐Maillart et al., 2016). Such interventions include the self‐identification of warning signs (e.g., critical situations) and coping strategies (e.g., goal‐directed responses) with the support of a clinician or therapist. However, they do not require people to link the two components together. This is known to compromise behaviour‐change (Armitage, 2008). Volitional help sheets could, therefore, support these existing self‐harm planning interventions by providing a means of helping people identify and link warning signs with effective coping strategies.

### Methodological considerations

Methodological features of this study need to be considered when interpreting the results. First, the study focused on self‐harm irrespective of motivation, consistent with many studies (Armitage et al., 2016; Rasmussen et al., 2016) and UK guidelines for the management and prevention of self‐harm (NICE, 2022). However, in the self‐harm literature, some researchers have distinguished self‐harm with suicidal intent from self‐harm without suicidal intent. Despite overlapping, these are theoretically and empirically independent behaviours (Butler & Malone, 2013). Research testing the extent to which implementation intention interventions can reduce these two types of self‐harm behaviours, separately, is therefore warranted. Nevertheless, focusing on self‐harm irrespective of motivation can be considered a strength of the study because it enables greater generalizability.

Second, self‐reported measures of behaviour were used. Such measures are potentially susceptible to affective (Watkins et al., 1996), cognitive (Fulcher, 2003) and self‐presentational (Paulhus, 2002) biases. However, the findings are held with confidence. All outcome measures were self‐reported and the significant effects were specific to the measure of behaviour in specified critical situations, in line with expectations. Additionally, in the social sciences, self‐report is recognized as a valuable method for measuring behaviour in research on self‐harm (Melson & O'Connor, 2019), suicidal ideation (Moscardini et al., 2021) and many other behaviours (Lansdown et al., 2021). A randomized controlled design was also used, meaning that any potential biases in self‐reported measures would have been equalized across the conditions. Furthermore, both conditions received an intervention, which reduces the possibility that demand effects accounted for the findings (Rosenthal, 1966). Meta‐analytic evidence also shows that implementation intentions generate similar‐sized changes in self‐report behaviour measures as they do in objective measures (Gollwitzer & Sheeran, 2006).

Third, the implementation intention intervention was tested over a three‐month period. Although a longer period may demonstrate longer‐term intervention effects, it should be noted that many studies have tested implementation intentions over shorter period that the one used in this study (Carrero et al., 2019). In addition, implementation intention effects have been shown to increase over time (Sheeran & Orbell, 1999).

Finally, the implementation intention intervention used in this study was administered to the general adult population within the community (aged 18+). Future research is needed to test the effectiveness of the intervention at reducing self‐harm in other groups. In particular, previous research has shown that many critical situations, which can trigger self‐harm, are similar in adolescent and adult cohorts (McManus et al., 2016; Scoliers et al., 2009). However, there are some situations (e.g., problems with schoolwork or being bullied) which have greater relevance to adolescent self‐harm (Madge et al., 2011). Further research with adolescents is therefore required, not only to test the effectiveness of the present intervention in this specific cohort, but also to co‐design and refine its content prior to administration. Including individuals with lived experience at the development stage of an implementation intention intervention would be beneficial as it has been found to lead to the identification of additional critical situations and goal‐directed responses (Keyworth et al., 2021) with the final version of the intervention rated positively by participants (Keyworth et al., 2021, 2022).

## CONCLUSIONS

This study provides the first evidence that implementation intentions can reduce self‐harm in the community. The intervention reduced self‐harm in the critical situation specified in people's implementation intentions for those with the required goal intentions to avoid self‐harm and for those at the greatest risk as indicated by frequently experienced self‐harm and suicide related mental images. Future research is needed to provide a controlled test of whether reductions in self‐harm can generalize from specified critical situations to contextually similar situations. Research is also needed to test the extent to which implementation intentions can reduce self‐harm in a community sample with and without suicidal intent, separately.

## AUTHOR CONTRIBUTIONS

**Abigail Paterson:** Conceptualization; data curation; formal analysis; funding acquisition; investigation; methodology; project administration; resources; software; validation; visualization; writing – original draft; writing – review and editing. **Mark A. Elliott:** Conceptualization; formal analysis; funding acquisition; methodology; project administration; resources; supervision; writing – review and editing. **Susan Rasmussen:** Conceptualization; funding acquisition; methodology; project administration; resources; supervision; writing – review and editing. **Louise A. Brown Nicholls:** Conceptualization; funding acquisition; methodology; project administration; resources; supervision; writing – review and editing.

## CONFLICT OF INTEREST STATEMENT

The authors declare that there are no potential conflicts of interest with respect to the research, authorship, and/or publication of this article.

## Supporting information


Appendix S1.

## Data Availability

The data that support this study are not publicly available due to ethical restrictions. The corresponding author may be contacted with any requests to run specific statistical analyses.

## Endnotes

^1^
The data that support this study are not publicly available due to ethical restrictions. The corresponding author may be contacted with any requests to run specific statistical analyses.

^2^
The intention to treat procedure used in the data analysis ensured that the achieved sample (*n* = 469) exceeded the required sample reported in the ‘participants’ section (*n* = 259). The analyses were therefore sufficiently powered. However, given the sample of study completers (*n* = 216) was lower than the required sample, all the ANCOVAs presented in Table 3 were re‐run as ANOVAs. Power analyses indicted that these ANOVAs required a sample of *n* = 204. Both the intention‐to‐treat sample and the sample of study completers exceeded this requirement, and the ANOVA findings were the same as the ANCOVA findings presented in Table 3 (i.e., there were still no significant differences between the experimental and control conditions on any outcome measure). Also note that the target sample reported in the ‘participants’ section was the largest sample that was required for any of the reported analyses. These were the ANCOVAs. A power analysis indicated that a sample of *n* = 114 was required for moderated linear regressions, which are reported later in the text. Both the intention‐to‐treat sample and the sample of study completers exceeded this requirement.
