# Supplementary material for: Evidence that implementation intentions reduce self‐harm in the community
Source: Br J Health Psychol. 2023 Aug 7;28(4):1241–60. doi: 10.1111/bjhp.12682 (PMC10947202; doi:10.1111/bjhp.12682)
Supplement: Supplementary file 1 — Appendix S1. [file BJHP-28-1241-s001.docx]

Appendix 1: Volitional Help Sheet

Research shows that individuals sometimes find themselves self-harming, even though they may not set out to do so. Research also shows that people are more likely to avoid self-harming if they identify:

(a) the situations in which they are likely to be tempted to self-harm and

(b) the strategies that they will use to overcome the temptation.

We would like you to do this now for four "tempting" situations and strategies, using the drop-down lists on the next pages.

| If I am tempted to self-harm when… | Then I will… |
| --- | --- |
| I want to get relief from a terrible state of mind | Remind myself about the benefits of not self-harming |
| I want to punish myself | Recall all that I know about the dangers of self-harming |
| I want to die | Do something instead of self-harming (e.g., doing some exercise, squeeze an ice cube, draw on yourself in red pen) |
| I want to show how desperate I am feeling | Take prescribed medication to stop me feeling this way |
| I want to find out whether someone really loves me | Tell myself that I can avoid self-harming if I want to |
| I want to get some attention | Remember that I have made a commitment not to self-harm |
| I want to frighten someone | Tell myself that Society is changing in ways that make it easier for people to avoid self-harming |
| I want to get my own back on someone | Remind myself that there are groups in society that now provide support to people who feel this way (e.g., Samaritans, the National Self-Harm Network Forum, Harmless) |
| I feel hopeless | Make sure I am rewarded by others if I don’t self-harm |
| I feel trapped by a situation (e.g., work, a relationship, obligations) | Make sure I reward myself if I don’t self-harm |
| I feel trapped inside myself by my own thoughts and feelings | Think about the impact my self-harming would have on the people around me |
| I feel powerless | Think about how harming myself might affect how people view me |
| I feel like I have lost my standing in the world | Remember that I get upset when I think about harming myself |
| I feel that I don’t belong | Think about the guilt or shame that I might feel after harming myself |
| I feel lonely | Tell myself that any feelings of relief due to harming myself will only be temporary |
| I feel people I like don’t like me back | Remember that there are people in my life who care for me |
| I have no one to turn to | Put things around my home or place of work (e.g., photographs of friends or loved ones, or reminders of happy times) that help me manage these feelings |
| I feel like I am a burden | Try to avoid putting myself in situations that make me feel this way in the future |
| I feel like others would prefer me not to be here | Seek out someone trustworthy who I can talk to about these feelings |
| I hate myself | Contact a helpline (e.g., Samaritans) or a self-harm support group |
|  | Tell myself that I do not deserve to be hurt |
|  | Remind myself that these feelings do not define who I am, and they do not mean I need to harm myself |
